# A genome-wide association study reveals cytokinin as a major component in the root defense responses against *Ralstonia solanacearum*

**DOI:** 10.1093/jxb/eraa610

**Published:** 2021-01-21

**Authors:** Alejandro Alonso-Díaz, Santosh B Satbhai, Roger de Pedro-Jové, Hannah M Berry, Christian Göschl, Cristiana T Argueso, Ondrej Novak, Wolfgang Busch, Marc Valls, Núria S Coll

**Affiliations:** 1 Centre for Research in Agricultural Genomics (CSIC-IRTA-UAB-UB), Bellaterra, Barcelona, Spain; 2 Gregor Mendel Institute (GMI), Austrian Academy of Sciences, Vienna Biocenter (VBC), Dr Bohr-Gasse 3, Vienna 1030, Austria; 3 Salk Institute For Biological Studies, Plant Molecular and Cellular Biology Laboratory, 10010 N Torrey Pines Rd, La Jolla, CA 92037, USA; 4 Department of Bioagricultural Sciences and Pest Management, Colorado State University, Fort Collins, CO 80523, USA; 5 Cell and Molecular Biology, Colorado State University, Fort Collins, CO 80523, USA; 6 Laboratory of Growth Regulators, Olomouc, The Czech Republic; 7 Genetics Department, University of Barcelona, Barcelona, Spain; 8 University of Edinburgh, UK

**Keywords:** Bacterial wilt, cytokinin, defense, GWAS, hormones, immune system, *Ralstonia solanacearum*, root, salicylic acid

## Abstract

Bacterial wilt caused by the soil-borne pathogen *Ralstonia solancearum* is economically devastating, with no effective methods to fight the disease. This pathogen invades plants through their roots and colonizes their xylem, clogging the vasculature and causing rapid wilting. Key to preventing colonization are the early defense responses triggered in the host’s root upon infection, which remain mostly unknown. Here, we have taken advantage of a high-throughput *in vitro* infection system to screen natural variability associated with the root growth inhibition phenotype caused by *R. solanacearum* in Arabidopsis during the first hours of infection. To analyze the genetic determinants of this trait, we have performed a genome-wide association study, identifying allelic variation at several loci related to cytokinin metabolism, including genes responsible for biosynthesis and degradation of cytokinin. Further, our data clearly demonstrate that cytokinin signaling is induced early during the infection process and cytokinin contributes to immunity against *R. solanacearum*. This study highlights a new role for cytokinin in root immunity, paving the way for future research that will help in understanding the mechanisms underpinning root defenses.

## Teaser

Using genome-wide association analysis, we identify cytokinin as an important component in early root defense responses against the bacterial wilt pathogen *Ralstonia solanacearum.*

## Introduction

Plant hormones are extremely important for the regulation of the plant defense against pathogens (Pieterse *et al.*, 2012). Several studies have shown that the accumulation of salicylic acid (SA) induces plant defense against biotrophic pathogens (Shigenaga and Argueso, 2016), whereas jasmonic acid (JA) and ethylene are essential against necrotrophs. The crosstalk between JA and abscisic acid (ABA) induces plant defense against herbivores and insects (Pieterse *et al.*, 2012). The synergistic or antagonistic interaction between the different hormone signaling pathways enables the plant to fine-tune defense responses to the pathogen that are effective while minimizing damage or yield penalties (Pieterse *et al.*, 2009).

Cytokinin is a plant hormone traditionally associated with plant growth and development (Mok, 1994; Sa *et al.*, 2001; Wybouw and De Rybel, 2019) with an emerging role in plant immunity. Cytokinin has been shown to participate in defense against various plant pathogens, including fungi (Argueso *et al.*, 2012; Gupta *et al.*, 2020), bacteria (Choi *et al.*, 2010; Naseem *et al.*, 2012; Pieterse *et al.*, 2012). and viruses (Clarke *et al.*, 1998a; Pogány *et al.*, 2004). Furthermore, application of exogenous cytokinin results in an increase of callose production in *Arabidopsis thaliana* (henceforth, Arabidopsis) infected with *Pseudomonas syringae* or treated with the flagellin-derived defense elicitor flg22 (Choi *et al.*, 2010). Tight regulation of cytokinin levels is essential to determine the precise signaling outcome. Treatments with low concentrations of exogenous cytokinin result in greater susceptibility to infection with the oomycete *Hyaloperonospora arabidopsidis* in Arabidopsis, and also to infection with *Blumeria graminis* in wheat. In contrast, treatments with higher levels of cytokinin increase resistance of plants to these and other pathogens (Argueso *et al.*, 2012; Babosha, 2009; Gupta *et al.*, 2020).

Importantly, cytokinin signaling in immunity is greatly intertwined with SA signaling. It has been observed that increased resistance to *H. arabidopsidis* induced by cytokinin treatment is mediated by SA accumulation and the activation of SA-dependent defense genes (Choi *et al.*, 2010; Argueso *et al.*, 2012). Mechanistically, it has been shown that the ARR2 (ARABIDOPSIS RESPONSE REGULATOR 2), a major transcription factor of the cytokinin signal transduction pathway, physically interacts with TGA3, a transcription factor from the SA signaling pathway (Choi *et al.*, 2010). The interaction of these two transcription factors is regulated by NPR1 (NON-EXPRESSOR OF PATHOGENESIS-RELATED PROTEINS 1), leading to changes in *PR1* expression and plant immune status (Choi *et al.*, 2010). More recently, it has been shown that cytokinin treatment of tomato leaves induces resistance against fungi in an SA-dependent manner (Gupta *et al.*, 2020). Although sparse, current evidence indicates that the crosstalk between cytokinin and SA signaling pathways is very important for plant immune responses (Choi *et al.*, 2010; Argueso *et al.*, 2012).

The majority of studies analyzing the role of cytokinin in plant defense have been performed using foliar pathogens (Clarke *et al.*, 1998a; Pogány *et al.*, 2004; Choi *et al.*, 2010; Argueso *et al.*, 2012; Naseem *et al.*, 2012; Pieterse *et al.*, 2012; Gupta *et al.*, 2020). In contrast, the role of cytokinin in root defenses remains mostly unexplored.

*Ralstonia solanacearum* is a natural soil-borne bacterial vascular pathogen that infects many plant species, including Arabidopsis, and it is the causative agent of bacterial wilt, a disease of devastating economic impact worldwide*. Ralstonia solanacearum* invades plants through the roots, moving centripetally until it reaches the xylem. Xylem colonization allows movement of the bacteria up into the stem, causing a rapid and permanent obstruction of the vasculature (Hayward, 1991; Planas-Marquès *et al.*, 2020). Many genetic tools are available to study this pathogen, since it has been widely used as a model species for plant–pathogen interactions in the last decades (Mansfield *et al.*, 2012; Coll and Valls, 2013).

Transcriptomic analyses of Arabidopsis roots infected with the bacterial pathogen *R. solanacearum* show expression of cytokinin biosynthetic genes at early time points [6 hours post-infection (hpi)] (Zhao *et al.*, 2019), while cytokinin-degrading genes are expressed at later stages of infection (after 72 h) in *Medigaco truncatula* roots infected with *R. solanacearum* (Moreau *et al.*, 2014). Interestingly, Arabidopsis plants lacking the redundant negative regulator of cytokinin signaling ARR6 showed increased resistance to the fungal pathogen *Plectosphaerella cucumerina* but increased susceptibility to *R. solanacearum* (Bacete *et al.*, 2020). However, these phenotypes did not occur as a direct result of the interactions between cytokinin signaling and classical hormone-based defense pathways.

In previous work, we set up an *in vitro* system to study Arabidopsis early root phenotypes caused by *R. solanacearum* infection: root growth inhibition, root hair formation, and root tip cell death (Lu *et al.*, 2018). This robust method revealed genetic determinants of the interaction both from the bacterial virulence and plant defense sides at very early stages of infection, which were masked in classic pathogenicity assays. Here, we have taken advantage of the high-throughput potential of this *in vitro* system to screen the natural variation of the root growth inhibition phenotype across 430 Arabidopsis accessions representative of the worldwide genetic variation of this species and determine the gene(s) responsible for this trait using genome-wide association (GWA) mapping.

Thanks to the large number of Arabidopsis accessions that have been sequenced and genotyped, this model plant has great potential for genome-wide association study (GWAS) analyses (Atwell *et al.*, 2010). Previous studies using GWAS and natural genetic variation have detected genetic variants associated with resistance to abiotic stress (Bac-Molenaar *et al.*, 2015; Kalladan *et al.*, 2017; Satbhai *et al.*, 2017; Li *et al.*, 2019), root development (Meijón *et al.*, 2014), or flowering time (Aranzana *et al.*, 2005). GWAS has also been shown to be a very powerful tool to unravel genomic regions associated with the natural variation of disease resistance of various plants against different pathogens, for example Arabidopsis against *Pseudomonas syringae* (Aranzana *et al.*, 2005; Atwell *et al.*, 2010; Iakovidis *et al.*, 2016), and *Xanthomonas campestris* (Huard-Chauveau *et al.*, 2013) and *Botrytis cinerea* (Corwin *et al.*, 2016; Thoen *et al.*, 2017), or *Glycine max* against *Fusarium virguliforme* (Wen *et al.*, 2014), among others. Importantly, a GWAS has been recently used to study the temperature-dependent genetic variation that underscores resistance of Arabidopsis against *R. solanacearum* (Aoun *et al.*, 2017). Finally, GWAS has also highlighted the importance of hormonal crosstalk between SA and ABA in the JA pathway involved in defense in Arabidopsis (Proietti *et al.*, 2018). Taking advantage of GWAS, we have identified cytokinin signaling as an important component in the root growth inhibition phenotype caused by *R. solanacearum* in Arabidopsis, contributing to root defenses against the pathogen.

## Materials and methods

### Plant material

A collection of 430 *A. thaliana* ecotypes (Supplementary Table S1) provided by the Molecular Plant Biology Stock from the Gregor Mendel Institute (Vienna, Austria) was used for GWAS. The Arabidopsis mutant lines used in this study have been previously described in Kiba *et al.* (2013) (*cyp735a1* and *cyp735a2*) and Caesar *et al.* (2011) (*ahk2*, *ahk3*, and *ahk4/cre1*). Transgenic line *TCSn::GFP* has been described in Zürcher *et al.* (2013). The *eds16 TCSn::GFP* line was obtained by crossing the *eds16* mutant (Dewdney *et al.*, 2000) to the TCSn::GFP transgenic line and screening F_2_ plants for the presence of *TCSn::GFP* by selection on Murashige and Skoog (MS) plates supplemented with BASTA and for *eds16* using PCR primers that can detect the *eds16* mutation (*eds16 Fwd*, CCTGAGAGACTATTCCAAAGGAC; *eds16 Rev*, ACTCTGAAGATGGGTCACTTCC). Homozygous seeds were used in all the assays.

### Plant and bacterial growth conditions for GWAS

Seeds were surface sterilized for 2 h in open 1.5 ml Eppendorf tubes in a sealed box containing chlorine gas generated from 125 ml of 10% w/v sodium hypochlorite and 3.5 ml of 37% hydrochloric acid. For stratification, sterile seeds were kept at 4 °C for 72 h in the dark. After that, seeds were put on agar plates containing MS (Duchefa Biochemie B.V., Haarlem, The Netherlands) and 0.8% agar (Becton, Dickinson and Co., Franklin Lakes, NJ, USA). The placement of the seeds was guided by a printout of a seed-planting grid schematic (Supplementary Fig. S1) placed below the plate. Each plate contained two accessions with six seeds per accession. To account for positional effects within and between the Petri dishes, we plated 12 seeds for each accession over two plates in a permutated block design. Plates were positioned in racks that oriented the plates in a vertical position to a growth chamber constantly kept at 21 °C and a 16 h light/8 h dark cycle, with a light intensity of 120 µmol m^–2^ s^–1^ during the light period. Plants were inoculated as described in the section below ‘*In vitro* inoculation assays’.

### Image acquisition

Root images were obtained using CCD flatbed scanners (EPSON Perfection V600 Photo, Seiko Epson Co., Nagano, Japan). The BRAT (Busch-lab Root Analysis Toolchain) image acquisition tool on a standard desktop computer running Ubuntu Linux allowed the simultaneous control of the scanners (Slovak *et al.*, 2014). Scans were performed with a resolution of 1200×1200 dpi, resulting in an image size of 6000×6000 pixels (36 MP) for each of our 12×12 cm agar plates. To enhance image quality, scanning was performed in a dark room and with the scanner lid open.

### Genome-wide association mapping

We measured median and mean total root length values of 430 Arabidopsis accessions after *R. solanacearun* infection using BRAT (*n*=2) to conduct GWA using an accelerated mixed model (EMMAX) (Kang *et al.*, 2010) followed by EMMA (Kang *et al.*, 2008) for the most significant associations among all accessions studied The GWA was performed on a cluster, with algorithms identical to those used in the GWAPP Web interface (Seren *et al.*, 2012). Single nucleotide polymorphisms (SNPs) with minor allele counts ≥10 were considered. The significance of SNP associations was determined at a 5% false discovery rate (FDR) threshold computed by the Benjamini–Hochberg–Yekutieli method (Benjamini and Hochberg, 1995).

### Broad-sense heritability calculation

All individuals that were measured were used to calculate the broad-sense heritability (H^2^=VG/VP), which is defined as the proportion of phenotypic variation (VP) due to genetic variation (VG) (estimated from the between-line phenotypic variance).

### Gene Ontology analysis

The GO-finder website (https://go.princeton.edu/) was used for Gene Ontology (GO) analysis. Genes solely ‘inferred from electronic annotation associations’ were excluded from the analysis.

### *In vitro* inoculation assays

Seeds were surface sterilized with a solution containing 30% bleach and 0.02% Triton X-100 for 10 min, washed five times with Milli-Q water, and sown (20 seeds per plate) on agar plates containing MS (Duchefa Biochemie B.V.) and 0.8% agar (Becton, Dickinson and Co.). Sown plates were stratified at 4 °C in the dark for 2 days. Plates were then transferred to chambers and grown vertically for 7 d under constant conditions of 21–22 °C, 60% humidity, and a 16 h light/8 h dark photoperiod with a light intensity of 120 µmol m^–2^ s^–1^ during the light period.

*Ralstonia solanacearum* GMI1000 was grown at 28 °C in solid or liquid rich B medium (0.1% yeast extract, 1% bacto pectone, and 0.1% casamino acids) (Becton, Dickinson and Company). For inoculation, *R. solanacearum* GMI1000 was collected by centrifugation (1500 rcf, 5 min) from overnight liquid cultures at 28 °C, resuspended with water, and adjusted to a final OD_600_ of 0.001 corresponding to 10^6^ colony-forming units (CFU) ml^–1^. Arabidopsis seedlings grown on plates as detailed above were inoculated with 5 µl of the bacterial solution, which was applied 1 cm above the root tip, as described previously (Digonnet *et al.*, 2012). Plates were then sealed with micropore tape (3M Deutschland GmbH, Neuss, Germany) and transferred to a controlled growth chamber at 25 °C, 60% humidity, and a 12 h light/12 h dark photoperiod with a light intensity of 120 µmol m^–2^ s^–1^ during the light period.

For the analysis of root growth inhibition and root hair formation, pictures were taken 48–72 hpi with an Olympus DP71 stereomicroscope (Olympus, Center Valley, PA, USA) at ×11.5. To analyze green fluorescent protein (GFP) root expression, roots from seedlings grown on plates were collected 48 hpi and photographed with a Leica DM6 epifluorescence microscope (Leica, Wetzlar, Germany). In order to quantify GFP fluorescence, the Leica Application Suite X (LAS X) software was used. A 0.1 cm section of the maturation zone was selected and GFP intensity was quantified as relative units and presented as the average of all roots measured. Three independent biological replicates were performed and, for each replica, 24 (Fig. 2) or 10 (Fig. 5) roots per condition were used.

### Exogenous cytokinin and salicylic acid application

For *R. solanacearum in vitro* root inoculation assays that included exogenous application of hormones, 7-day-old seedlings were transferred from MS agar plates to fresh MS agar plates supplemented with different hormone concentrations (25 nM and 50 nM kinetin; 1.5 µM and 7.5 µM SA) from Duchefa Biochemie. Roots were inoculated 24 h later as described above.

### Pathogenicity assays

*Ralstonia solanacearum* pathogenicity tests were carried out using the soil-drench method (Monteiro *et al.*, 2012). Briefly, Arabidopsis was grown for 4 weeks in Jiffy pots (Jiffy Group, Lorain, OH, USA) in a controlled chamber at 22 °C, 60% humidity, and an 8 h light/16 h dark photoperiod. Jiffys were drilled three times with a wooden stick and immediately submerged for 30 min into a solution of overnight-grown *R. solanacearum* adjusted to OD_600_=0.1 corresponding to 10^8^ CFU ml^–1^ with distilled water (35 ml of bacterial solution per plant). Inoculated plants were transferred to trays containing a thin layer of soil drenched with the same *R. solanacearum* solution and kept in a chamber at 27 °C, 60% humidity, and 12 h light/12 h dark. Plant wilting symptoms were recorded every day and expressed according to a disease index scale (0, no wilting; 1, 25% wilted leaves; 2, 50%; 3, 75%; and 4, death) (Supplementary Fig. S5). At least 30 plants per condition were used in each assay, and at least three replicates were performed for every experiment.

### Quantitative reverse transcription–PCR (RT–qPCR)

Roots were collected from *R. solanacearum-*infected or water-treated Arabidopsis plants at 0, 24, and 48 hpi. Briefly, roots from ~40 seedlings were cut and pooled. Roots were rapidly washed in water and dried before freezing in liquid nitrogen. Samples were stored at –80 °C. RNA was extracted using the Maxwell 16 LEV Plant RNA Kit (Promega, Australia) according to the manufacturer´s recommendations. RNAs were treated with DNase-free RNase (Promega, Australia) and the concentration measured with an ND-8000 Nanodrop. cDNA was synthesized from 2 µg of RNA using the High Capacity cDNA Reverse Transcription Kit (Applied Biosystems, USA) according to the manufacturer’s instructions. According to the SYBR Green PCR mix instructions (Roche, Switzerland), 2.5 µl of cDNA were used in a final reaction volume of 10 µl in the LightCycler 480 System (Roche, Switzerland). Melting curves and relative quantification of target genes were determined using the software LightCycler V1.5 (Roche, Switzerland). The amplification program was set to an initial step of 10 min at 95 °C followed by 45 cycles using 95 °C for 10 s, 60 °C for 30 s, and 72 °C for 30 s. All samples were run in triplicate for each biological replicate, and the target gene was normalized to the endogenous control gene Arabidopsis tubulin β-1 chain (*At1g75780*). To visualize the data, we calculated the fold change of each biological replicate in 24 h and 48 h samples by normalizing to the ΔCt of time point 0 hpi of the mock and infected samples separately. The statistical analysis of the normalized data was performed using the ‘rstatix’ R package (ver. 0.6.0). To test for differences in gene expression between mock and infected samples, the normalized data were tested for normality and homogeneity of variances. If these two requirements were fulfilled, the parametric *t*-test was performed for each time point to compare between mock and infected samples. All primer sequences used were obtained from previous publications and are listed in Supplementary Table S6. qPCR analysis conforms to the Minimum Information for Publication of Quantitative Real-Time PCR Experiments (MIQE) guidelines (Bustin *et al.*, 2009).

### Cytokinin analysis (LC-MS/MS)

Arabidopsis plants were grown in pots with sand for 5 weeks in a controlled chamber at 22 °C, 60% humidity, and an 8 h light/16 h dark photoperiod, with a light intensity of 120 µmol m^–2^ s^–1^ during the light period. The sand in the pots was drilled three times with a wooden stick and immediately irrigated with bacterial solution of overnight-grown *R. solanacearum* adjusted to OD_600_=0.1 with distilled water (50 ml of bacterial solution per plant). Trays with plant pots infected were transferred to a chamber at 27 °C, 60% humidity, and 12 h light/1 2h dark. Then, at 4–7 days post-inoculation (dpi), inoculated roots were washed with distilled water and dried with filter paper. After that, the root samples were weighed and stored at –80 °C. Four biological replicates with 20 mg each were used each time (0, 4, and 7 dpi). Cytokinin levels were measured as described previously (Poitout *et al.*, 2018).

## Results

### Genome-wide association mapping reveals several loci associated with cytokinin metabolism in Arabidopsis roots infected with *R. solanacearum*

Infection of Arabidopsis roots with *R. solanacearum* GMI1000 *in vitro* results in root growth inhibition. We previously observed natural variation of this phenotype across a small population of Arabidopsis accessions (Lu *et al.*, 2018). To identify loci responsible for this natural variation, we performed a GWAS using a collection of 430 Arabidopsis accessions representative of the worldwide genetic variation of this species (Supplementary Fig. S1; Supplementary Table S1). Arabidopsis seeds were sown on agar plates following the scheme presented in Supplementary Fig. S1 to ensure randomization. After 7 d, seedlings were inoculated 1 cm over the root tip with a 5 µl droplet of a 10^6^ CFU ml^–1^ suspension of *R. solanacearum* GMI1000. Images of seedlings were then acquired using scanners every day for 5 d to measure root length and to monitor the impact on root growth caused by *R. solanacearum* infection *in vitro*. Differences in root length between accessions were monitored after infection and subsequently analyzed.

**Fig. 1.**
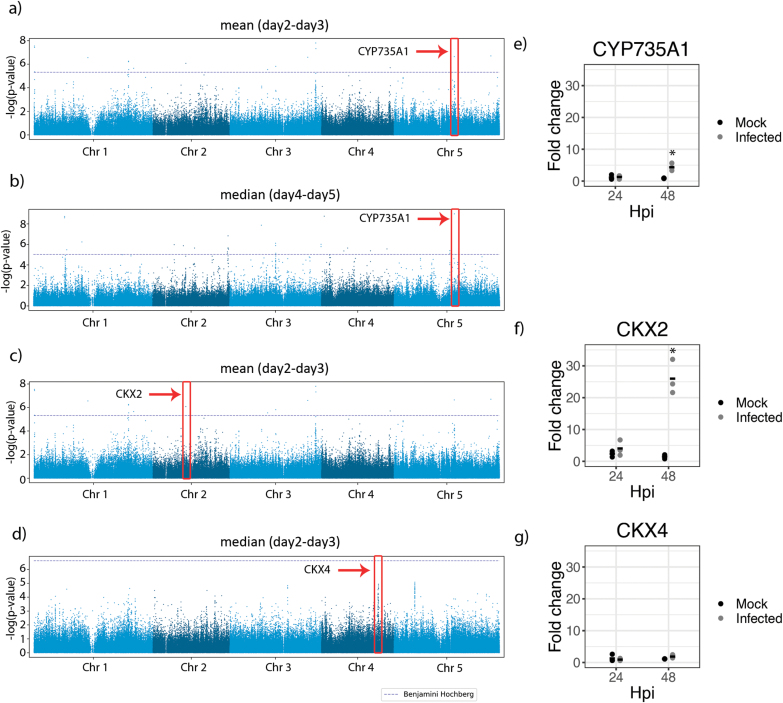
GWA analysis reveals association of cytokinin metabolism genes with root growth inhibition caused by *R. solanacearum* infection of Arabidopsis roots. (A–D) Manhattan plots of GWA results for root growth traits. Different colors represent different chromosomes. The horizontal dashed lines correspond to a nominal *P*<0.05 significance threshold after Benjamini–Hochberg correction. Solid red boxes highlight the SNPs with the highest *P*-values in: (A) mean relative root growth rate between day 2 and day 3 (*P*-value 2.42E-07); (B) median root growth rate between day 4 and day 5 (*P*-value 1.64E-09); (C) mean relative root growth rate between day 2 and day 3 (*P*-value 8.89E-07); and (C) median root growth rate between day 2 and day 3 (*P*-value 6.55E-07). Fold change values of the quantitative PCR analysis of (E) *CYP735A1*, (F) *CKX2*, and (G) *CKX4* using *TUBULIN* as control. Asterisks indicate statistically significant differences in a paired Student’s *t*-test (*P<0.05) between 48 hpi and normalizing by the values of the time point control (0 h hpi).

To identify sequence variation in genomic regions associated with the variation of the root growth inhibition phenotype caused by the *R. solanacearum* root infection, we conducted GWA mapping using the Arabidopsis 250K SNP chip data (Horton *et al.*, 2012) with a mixed model correcting for population structure (Seren *et al.*, 2012) and the root growth data described in Supplementary Table S2. Because we were interested in the root growth responses upon *R. solanacearum* root infection, we focused our analysis on root growth rates. The broad-sense heritability (H^2^) of these traits ranged from 10% to 55% with an average of 36% (Supplementary Table S3). We observed 20 unique SNPs significantly associated with the root growth responses to *R. solanacearum* infection using a 5% Benjamini–Hochberg threshold (Supplementary Table S4). The most significant of these associations (SNP 15401974, chromosome 5; *P*-value 1.64×10^–9^; FDR 5.6×10^–5^) was found for two root growth rate measurements: the mean of the relative root growth rate between day 2 and day 3 (Fig. 1A); and the median of the relative root growth rate between day 4 and day 5 (Fig. 1B). Because this SNP displayed the most significant *P*-value and was found in traits relating to two different days of the time course, we concluded that it might be important in explaining the root growth phenotypic variation between accessions. While this SNP is located within the 5 kb upstream region of multiple genes (*At5g38450* and *At5g38460*) (Supplementary Fig. S2), the highest level of linkage disequilibrium in any gene of this region with the top SNP can be observed for an SNP in the *At5g38450* gene (Pearson coefficient of correlation *r*=0.39). This gene encodes a cytokinin hydroxylase (*CYP735A1*) that catalyzes the biosynthesis of the cytokinin *trans-*zeatin (Takei *et al.*, 2004). Another analysis guided our focus towards the cytokinin pathway: when conducting a GO enrichment analysis of genes in 10 kb proximity to SNPs associated with root growth rate upon infection (EMMAX *P*-value <10^–6^) (Supplementary Table S4), we found the process cytokinin catabolism to be significantly enriched (*P*-value 0.00025; FDR 4.86%; Supplementary Table S5). These included two additional genes associated with cytokinin metabolism, the cytokinin oxidases *At2g19500* (*CKX2*) and *AT4G29740* (*CKX4*). *CKX2* is upstream of an SNP significantly associated with mean relative root growth rate between day 2 and day 3 (SNP 8436350; chromosome 2; *P*-value 8.89×10^–7^; FDR 0.015) (Fig. 1C) and *CKX4* is upstream of an SNP marginally associated with median root growth rate between day 2 and day 3 (SNP 14577216; chromosome 4; *P*-value 6.55×10^–7^; FDR 0.101) (Fig. 1D). Both genes code for proteins that catalyze the degradation of cytokinins (Mok and Mok, 2001).

Next, we analyzed the level of expression of *CYP735A1*, *CKX2*, and *CKX4* in Arabidopsis Col-0 roots. This ecotype was selected for further analysis because it has been widely used for pathogenicity assays using *R. solanacearum*, many genetic resources are available, and it is susceptible to the widely available GMI1000 strain, with a clearly observable root inhibition phenotype that appears at early stages of infection (Lu *et al.*, 2018; Supplementary Fig. S3). Quantitative PCR was used to compare plants infected with *R. solanacearum* and mock-treated plants at 0, 24, and 48 h post-treatment. *Ralstonia solanacearum* infection consistently induced expression of two of these three genes (*CYP735A1* and *CKX2*) at 48 hpi (Fig. 1E–G), indicating a potential involvement of cytokinin signaling in plant root defenses against this bacterial pathogen.

### Early *R. solanacearum* infection induces cytokinin signaling in Arabidopsis roots.

**Fig. 2.**
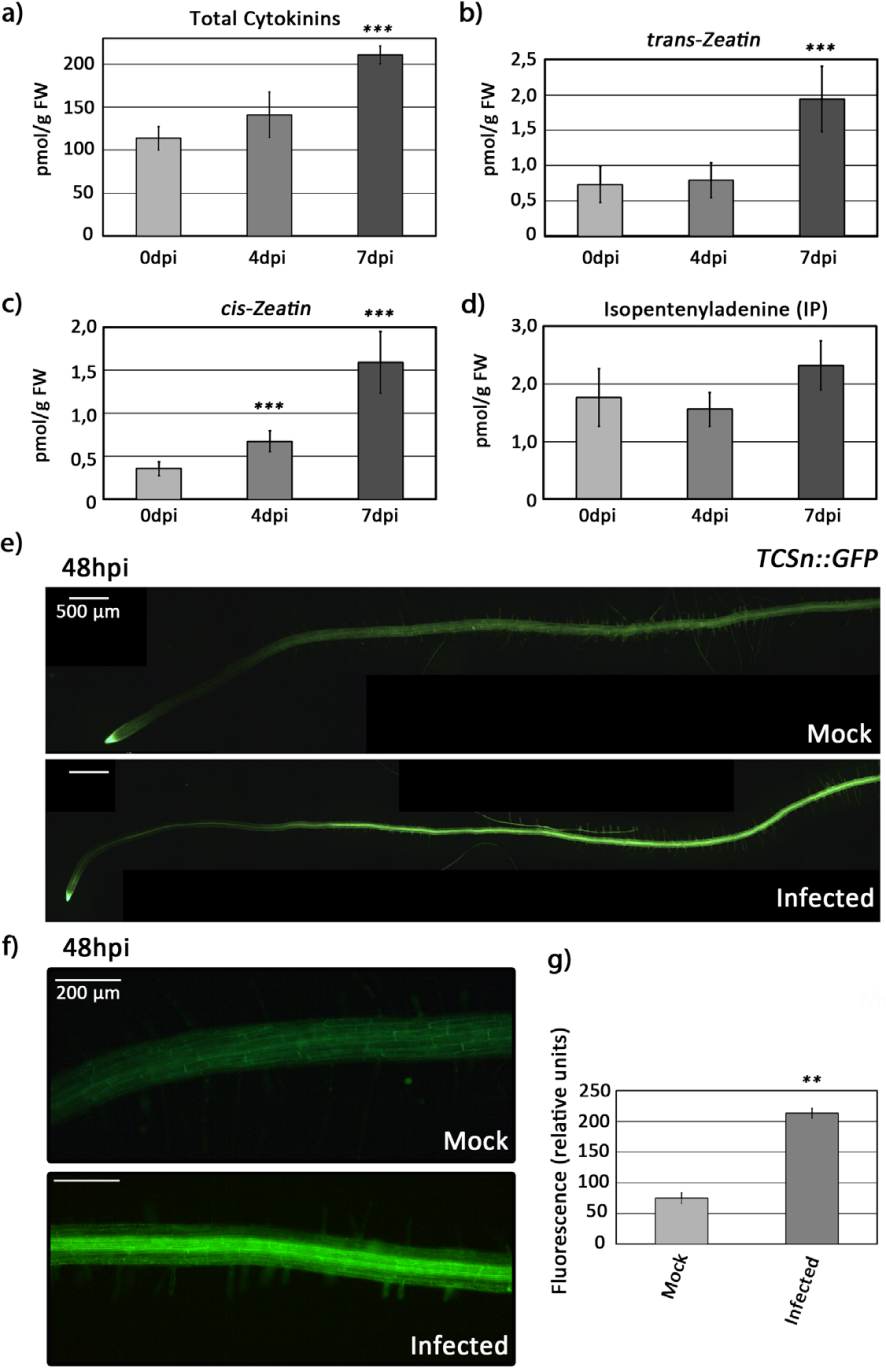
Early *R. solanacearum* infection induces cytokinin signaling in Arabidopsis Col-0 roots. Four-week-old Col-0 plants were inoculated with *R. solanacearum*, and (A) total cytokinin, (B) *trans-*zeatin, (C) *cis-*zeatin, and (D) isopentenyladenine concentrations were analyzed in inoculated root tissues at the indicated times (0, 4, and 7 dpi) by LC-MS/MS using four biological replicates. Error bars correspond to SEs. Asterisks indicate statistically significant differences between 4 and 7 dpi and the control (0 dpi) in a paired Student’s *t*-test (***P <0.001). (E–G) Six-day-old seedlings stably expressing the cytokinin signaling marker *TCSn::GFP* were inoculated with *R. solanacearum* or water, and roots at 48 hpi were observed under an epifluorescence microscope. *TCSn::GFP* signal in (E) whole roots and (F) the root maturation zone. (G) Quantification of the fluorescence intensity on the maturation zone (F) corresponds to the average GFP intensity from 24 individual roots per condition, calculated using the LAS X software. The experiment was repeated three times with similar results. Error bars correspond to SEs. Asterisks indicate statistically significant differences between 48 hpi and the control (0 hpi) in a paired Student’s *t*-test (**P<0.01).

Next, we analyzed whether *R. solanacearum* infection resulted in an increase of cytokinin content in Arabidopsis Col-0 roots. For this, we measured the levels the cytokinins *trans*-zeatin, *cis*-zeatin, and isopentenyladenine, as well as total cytokinins using LC-MS/MS. We could observe a significant increase in *trans*- and *cis-*zeatin, as well as total cytokinins after infection (Fig. 2A–D). We could only detect significant increases at later stages of infection (4 and 7 dpi), probably due to the sensitivity constraints of the measurement method.

In order to more specifically investigate the early effects of *R. solanacearum* infection on root cytokinin signaling, we took advantage of a more sensitive approach by analyzing expression of the synthetic Arabidopsis cytokinin reporter *TWO COMPONENT SIGNALING SENSOR* new (*TCSn*) fused to GFP (*TCSn::GFP*) (Zürcher *et al.*, 2013). Arabidopsis seedlings stably expressing *TCSn::GFP* were grown vertically on MS medium during 7 d and then roots were inoculated with *R. solanacearum* (see the Materials and methods). Infection resulted in a strong induction of *GFP* expression driven by the cytokinin signaling reporter *TCSn* in the vasculature of the root maturation zone (Fig. 2B, C). The intensity of the *GFP* induction caused by *R. solanacearum* infection at 48 hpi was four times higher than in the water control, which clearly indicated that cytokinin signaling is engaged in root responses to *R. solanacearum* invasion (Fig. 2D).

### Plants affected in cytokinin biosynthesis and perception display enhanced susceptibility towards *R. solanacearum*

**Fig. 3.**
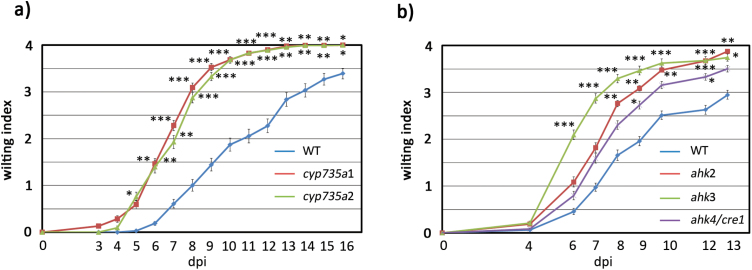
Cytokinin biosynthesis and perception are important for the plant response against *R. solanacearum* root infection. Four-week-old plants were soil-drench inoculated with *R. solanacearum* and symptoms were measured over time using a disease index on a scale of 1 to 4 (0=no wilting, 1=25% wilted leaves, 2=50%, 3=75%, and 4=death). (A) Wild-type Col-0 and *trans-*zeatin biosynthesis mutants *cyp735a1* and *cyp735a2*. (B) Wild-type Col-0 and cytokinin sensor histidine kinase mutant genes (*ahk2*, *ahk3*, and *ahk4/cre1*). Each experiment was repeated at least three times obtaining similar results, using 24 plants per experiment. Error bars correspond to SEs. Asterisks indicate statistically significant differences between the wild type and different mutant lines in a paired Student’s *t*-test (*P<0.05, **P<0.01, ***P<0.001).

If cytokinin levels and cytokinin signaling are important for root defense responses against *R. solanacearum*, it would be expected that impairment of cytokinin biosynthesis results in enhanced susceptibility to the pathogen. To address this question, we performed pathogenicity assays on knockout mutants of the cytokinin biosynthetic enzymes CYP735A1 and CYP735A2, which do not display any apparent phenotype (Kiba *et al.*, 2013). For this, 4-week-old Arabidopsis plants were inoculated with *R. solanacearum* GMI1000 by soil drenching, and symptoms were evaluated over time following a disease index scale (Lu *et al.*, 2018). Both *cyp735a1* and *cyp735a2* showed earlier wilting disease symptoms and were dramatically more susceptible to *R. solanacearum* than wild-type plants (Fig. 3A). This clearly indicates that cytokinin biosynthesis is involved in defense responses against *R. solanacearum*.

Based on this, we hypothesized that cytokinin perception would be equally important for immune responses against *R. solanacearum*. To test this idea, we performed pathogenicity assays on knockout mutants of the sensor histidine kinases AHK2, AHK3, and CRE1/AHK4, which act as cytokinin receptors (Ueguchi *et al.*, 2001; Higuchi *et al.*, 2004). All three cytokinin receptor mutants *ahk2*, *ahk3*, and *cre1/ahk4*, which grow normally on soil (Ueguchi *et al.*, 2001; Higuchi *et al.*, 2004), displayed enhanced susceptibility to *R. solanacearum* infection (Fig. 3B), indicating that perception of cytokinin is an important component of defense responses during *R solanacearum* infection.

### Exogenous cytokinin application partially reverts *R. solanacearum*-induced early root phenotypes

**Fig. 4.**
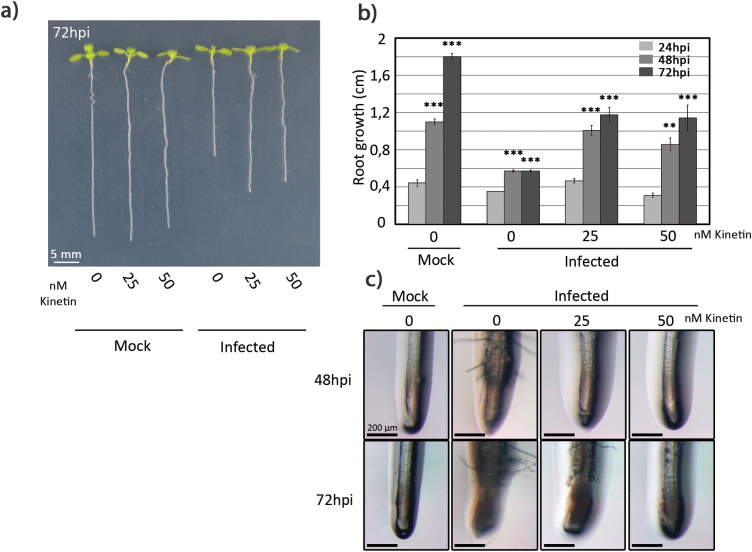
Exogenous cytokinin application partially reverts *R. solanacearum*-induced early root phenotypes. Seven-day-old Col-0 seedlings were grown for 24 h in MS medium supplemented with kinetin (0, 25, and 50 nM) and were inoculated with *R. solanacearum* or water. (A) An image of representative plants was obtained using a stereoscope 72 h after infection or mock treatment. (B) Root growth was measured at the indicated time points. (C) Images of representative roots were obtained using a stereoscope at the indicated time points after infection. Error bars correspond to SEs. Asterisks indicate statistically significant differences between 24, 48, and 72 hpi in a paired Student’s *t*-test (**P<0.01, ***P<0.001).

Our next goal was to determine whether exogenous cytokinin application could counteract the effects caused by *R. solanacearum* infection using the *in vitro* early root phenotypes as a measurable output (root growth inhibition and root hair production) (Lu *et al.*, 2018). For this, 7-day-old seedlings grown *in vitro* were transferred to fresh MS medium supplemented with different concentrations of the natural cytokinin kinetin (0, 25, and 50 nM). After 24 h, roots were pin-inoculated with *R. solanacearum* 1 cm above the root tip, and root growth inhibition and root hair production were monitored over time. Interestingly, kinetin supplementation (both 25 nM and 50 nM concentrations) resulted in partial reversion of the root growth inhibition phenotype caused by *R. solanacearum in vitro* (Fig. 4A, B). Whereas untreated inoculated seedlings stopped growing at 24 hpi, kinetin-treated inoculated seedlings kept growing, although to a lesser extent than non-infected roots. In addition, root hair production resulting from *R. solanacearum* infection was also inhibited by the kinetin pre-treatment (Fig. 4C). This effect was more pronounced when using a higher kinetin dose (50 mM), where no root hairs were observed. In contrast, the lower dose resulted in delayed but visible root hair production. We can thus conclude that kinetin pre-treatment at concentrations between 25 nM and 50 nM can alleviate the early root *in vitro* phenotypes caused by *R. solanacearum* infection, without causing toxicity to the plants. This indicates that cytokinin contributes to the onset of early responses that take place upon *R. solanacearum* infection in the root.

### Salicylic acid contributes to cytokinin signaling in Arabidopsis roots in response to *R. solanacearum* infection

**Fig. 5.**
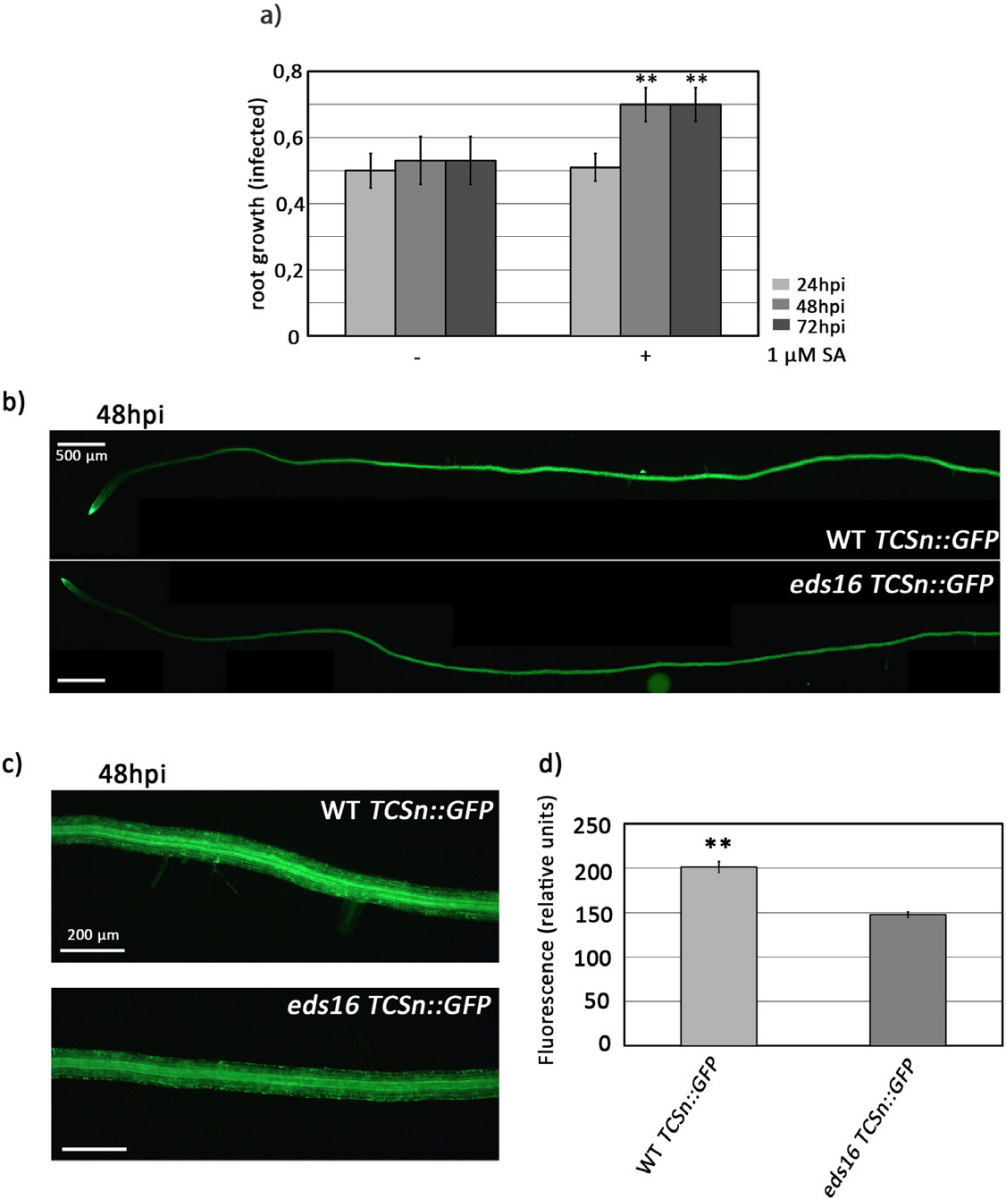
SA contributes to cytokinin signaling in roots in response to *R. solanacearum* infection. (A) Seven-day-old Col-0 wild-type seedlings were grown for 24 h in MS medium supplemented with SA (0 µM and 1 µM) and were then inoculated with *R. solanacearum*. Root growth was measured. Error bars correspond to SEs. Asterisks indicate statistically significant differences between 24, 48, and 72 hpi in a paired Student’s *t*-test (**P<0.01). (B–D) Six-day-old seedlings stably expressing the cytokinin signaling marker *TCSn::GFP* in the Col-0 and *eds16* background were inoculated with *R. solanacearum*, and at 48 hpi roots were observed under an epifluorescence microscope. *TCSn::GFP* signal in (B) whole roots and (C) the root maturation zone. This experiment was performed twice using 10 plants per genotype in each experiment. (D) Quantification of the fluorescence intensity on the maturation zone (C) corresponds to the average GFP intensity from 10 individual roots per condition calculated using the LAS X software. The experiment was repeated three times with similar results. Error bars correspond to the SE. Asterisks indicate statistically significant differences between 48 hpi and the control (0 hpi) in a paired Student’s *t*-test (**P<0.01).

A crosstalk between cytokinins and SA has been previously shown to regulate plant defenses against pathogens infecting leaves, such as *Pseudomonas syringae* (Choi *et al.*, 2010), *Hyalopenospora arabidopsidis* (Argueso *et al.*, 2012), *Botrytis cinerea*, and *Oidium neolycopersici* (Gupta *et al.*, 2020). However, whether a crosstalk between these hormones in root defenses takes place has not been determined. Previous *R. solanacearum* pathogenicity tests have not detected differences in susceptibility between wild-type and SA-deficient plants (*sid2* mutant or *NahG* transgenic lines, carrying an SA-degrading enzyme) (Hirsch *et al.*, 2002; Hernández-Blanco *et al.*, 2007; Hanemian *et al.*, 2016). However, exogenously applied SA had a clear effect on the root phenotypes induced by *R. solanacearum* infection *in vitro*. Seven-day-old seedlings were transferred for 24 h to MS medium supplemented with different SA concentrations (0, 1, 5, and 7.5 µM). We observed that SA concentrations >1 µM (5 µM and 7.5 µM) caused root growth inhibition of up to 50% of untreated seedlings (Supplementary Fig. S4A), as reported in previous studies (Pasternak *et al.*, 2019). Therefore, we performed *R. solanacearum in vitro* inoculation assays only on seedlings pre-treated with 1 µM SA, which did not cause any obvious effect on root growth before inoculation. Seven-day-old seedlings were transferred to MS medium supplemented with 1 µM SA and, 24 h later, roots were inoculated with *R. solanacearum* and monitored over time for root growth inhibition and root hair production. Exogenous application of 1 µM SA partially reverted the root inhibitory phenotype caused by *R. solanacerum* infection (Fig. 5A). On the other hand, 1 µM SA did not have any significant effect on root hair production (Supplementary Fig. S4b). Root hair production was partly inhibited only when higher SA concentrations (5, 7.5, and 1 µM) were exogenously supplied prior to *R. solanacearum* infection (Supplementary Fig. S2b). Considering that these concentrations affect root growth under normal conditions (Supplementary Fig. S2A), root hair inhibition may be a pleiotropic growth/development phenotype derived from SA toxicity rather than the result of SA modulation of defense responses to *R. solanacearum*.

To ascertain whether SA contributes to the cytokinin signaling involved in the response of Arabidopsis Col-0 roots to *R. solanacearum* root infection, we tested if impairing SA signaling would result in a decrease of cyokinin signaling outputs. For this, we quantified expression of *TCSn::GFP* in transgenic lines in a wild-type or an *eds16* mutant background, which is impaired in SA biosynthesis upon pathogen challenge (Dewdney *et al.*, 2000). *TCSn::GFP* expression after infection is reduced when SA signaling is suppressed, compared with the wild type. This can be observed at the whole-root level (Fig. 5B) and in a magnified area of the root maturation zone (Fig. 5C). Fluorescence quantification shows that in *eds16* mutant plants, *TCSn::GFP* expression is 25% lower than in a *TCSn::GFP* wild-type background (Fig. 5D). This indicates that SA signaling affects cytokinin signaling in response to *R. solanacearum* infection, pointing towards a potential cytokinin–SA crosstalk occurring in roots in response to infection with soil-borne pathogens. Further research in this area will clarify the possibility of a cytokinin–SA crosstalk during responses to pathogens in roots.

## Discussion

### Role of cytokinin in the interaction between *R. solanacearum* and Arabidopsis

In this study, we have taken advantage of GWAS to understand the genetic nature of the root phenotypic changes induced by *R. solanacearum* on Arabidopsis roots during early stages of infection. GWAS has been previously used to understand the basis of resistance against *R. solanacearum* in Arabidopsis under different temperatures and inoculation conditions (Aoun *et al.*, 2017). The study by Aoun and colleagues used wilt disease index rates over time as a trait to underscore temperature-dependent genetic diversity. At lower temperatures (27 °C) the main resistance quantitative trait locus (QTL) identified was RPS4/RRS1-R, a plant immune receptor pair with a very well-known role in resistance of Arabidopsis to *R. solanacearum* (Deslandes *et al.*, 2002; Le Roux *et al.*, 2015; Sarris *et al.*, 2015). In addition to that, this study revealed a new potential susceptibility gene at higher temperatures (30 ºC), strictosidine synthase-like 4 (SSL4), which encodes a protein with structural similarities to animal proteins involved in immunity (Aoun *et al.*, 2017). This study highlights the power of GWAS in revealing new potential sources of resistance to be engineered into crops.

Our study focuses on the same Arabidopsis–*R. solanacearum* pathosystem but from a different angle. At the very early stages of infection (2–3 dpi), *R. solanacearum* infection results in quick root growth inhibition, root hair formation, and root meristem cell death, which can be easily observed and screened in *in vitro* inoculation assays. We detected natural variation associated with these phenotypes among a small subset of accessions representative of Arabidopsis diversity (Lu *et al.*, 2018). Based on that observation and on the fact that the initial stages of plant colonization by *R. solanacearum* are poorly understood, despite being important for establishment of the bacteria inside the plant, we took advantage of GWAS to analyze the genetic diversity associated with one of these traits: root growth inhibition. Root hair formation and root meristem cell death were not included in GWAS because the technology at hand did not allow precise measurement of these traits.

Using GWAS, we screened root growth inhibition at different time points after infection on a large number of Arabidopsis accessions and focused on three candidate loci in the close proximity of SNPs that are significantly associated with this phenotype (Fig. 1A–C). These three genes are involved in the metabolism of cytokinin: *CYP735A1* in biosynthesis (Takei *et al.*, 2004), and *CKX2* and *CKX4* in cytokinin degradation (Mok and Mok, 2001). Gene expression analysis by qPCR showed that the expression of these genes in Col-0 roots was consistently induced by *R. solanacearum* at 48 hpi (Fig. 1E, F). Although the genes involved in cytokinin degradation, *CKX2* and *CKX4*, have not been investigated further in this work, they might participate in modulating the increased cytokinin levels in response to *R. solanacearum* infection.

Our data are in line with previous data underscoring a potential role for cytokinins in plant defense against *R. solanacearum*. RNA sequencing results show induction of genes involved in cytokinin synthesis (*CYP735A2*, *LOG2*, and *LOG6*), degradation (*CKX2*, *CKX3*, and *CKX5*), and response regulation (*ARR3*, *ARR4*, *ARR5*, *ARR7*, and *ARR16*) in Arabidopsis Col-0 roots at early time points after *R. solanacearum* infection (Zhao *et al.*, 2019). Moreover, genes controlling cytokinin metabolism (*LOG* and *CKX*), signaling (*ARR* genes). and perception (*CRE1*) have been shown to be differentially expressed in roots of the susceptible A17 *Medicago truncatula* genotype after infection with *R. solanacearum* (Moreau *et al.*, 2014). Importantly, Arabidopsis plants deficient in ARR6 show altered cell wall composition and are more susceptible to infection with *R. solanacearum* (Bacete *et al.*, 2020). Interestingly, Aoun *et al.* (2017) found two cytokinin-related genes among their top SNPs obtained upon infection with *R. solanacearum* at high temperatures (30 °C): the signal receptor AHK3 and the cytokinin response factor CRF2. In agreement with this, we have found that *ahk3* knockout mutants are more susceptible to *R. solanacerum* than the wild-type control (Fig. 3B).

Coupled to these increases in cytokinin-regulated gene expression, we observed an activation of cytokinin signaling in the root 48 hpi with *R. solanacearum*, as evidenced by expression of the reporter *TCSn::GFP* (Fig. 2E–G). Together, these data indicate that *R. solanacearum* triggers cytokinin production in the root by means of induction of gene expression of cytokinin biosynthetic genes, which is accompanied by activation of cytokinin signaling; in parallel, cytokinin degradation genes are up-regulated, to ensure a timely response and a tight regulation of cytokinin levels in the plant as has been described in the literature (Rashotte *et al.*, 2003; Brenner *et al.*, 2012).

Furthermore, we could observe an increase of cytokinin levels in the root after infection (Fig. 2A–D), albeit at later stages of infection, since at early stages reliable detection was challenging. When assessing which cytokinin forms were most abundant, we could detect that *R. solanacearum* resulted in an increase in both *trans-* and *cis-*zeatin levels, whereas the levels of isopentenyladenine did not show significant changes. The fact that *trans-*zeatin was among the most abundant forms was not surprising, since it is one of the most active forms of cytokinin in plants (Sakakibara, 2006). In contrast, *cis-*zeatin has always been regarded as an isomer with lower activity in plants. In fact, the study of *cis-*zeatin in the context of plant–pathogen interactions has only been addressed in the *Nicotiana tabacum–Pseudomonas syringae* pathosystem, where the exogenous addition of this isomer promotes the resistance of the plant against the pathogen (Groβkinsky *et al.*, 2011). Our data indicate that cytokinin may play a role in root defense against *R. solanacearum*, with *cis-* and *trans-*zeatin as two potentially important cytokinin forms for this defense function.

Taking advantage of the genetic resources available for Arabidopsis, we tested whether cytokinin synthesis and/or perception participated in defense against *R. solanacearum*. For this, we carried out pathogenicity tests, comparing a variety of mutants with defects in cytokinin perception (*ahk2*, *ahk3*, and *ahk4/cre1*) and biosynthesis (*cyp735a1* and *cyp735a2*). Our data clearly showed that both cytokinin synthesis and perception participate in defense against *R. solanacearum*, as defects in either pathway result in enhanced susceptibility towards the pathogen (Fig. 3).

Furthermore, application of low concentrations of the cytokinin kinetin partially reversed the phenotypes caused by *R. solanacearum* infection in Arabidopsis roots (Fig. 4). A plausible explanation could be that exogenous cytokinin application induces the expression of defense-related genes in the root, as we have shown here (Fig. 1F, G) similar to what has been previously reported for leaves (Rashotte *et al.*, 2003; Choi *et al.*, 2010; Argueso *et al.*, 2012). In fact, high doses of cytokinin were shown to induce resistance in Arabidopsis against the oomycete *H. arabidopsidis* (Argueso *et al.*, 2012), against *P. syringae* in Arabidopsis (Choi *et al.*, 2010), or even against virus replication in *Phaseolus vulgaris* (Clarke *et al.*, 1998b). In our root system, low doses of cytokinin were sufficient to partially prevent the root phenotypes caused by *R. solanacearum.* We did not use high doses of cytokinin because they have been shown to inhibit primary root (To *et al.*, 2004; Argyros *et al.*, 2008) growth.

### The impact of SA on the cytokinin signaling involved in the response of Arabidopsis roots to *R. solanacearum* infection

Previous research, performed mostly in shoot tissue, showed that the role of cytokinins in plant immunity is deeply related to SA signaling, with a clear crosstalk between the two signaling pathways taking place (Choi *et al.*, 2010; Argueso *et al.*, 2012; Gupta *et al.*, 2020). Choi *et al.* demonstrated that the SA-dependent TGA3 transcription factor binds to the response regulator ARR2, which is modulated by cytokinin signaling, to generate a complex that binds to the PR1 promoter and promotes defense against *P. syringae* (Choi *et al.*, 2010). Also, it has been shown that cytokinin regulates plant immunity against the oomycete *H. arabidopsidis* through the elevation of defense responses that are dependent on SA (Argueso *et al.*, 2012).

Here, we addressed whether SA had any impact on cytokinin signaling induced as part of the defense responses against root-invading pathogens and root immunity. Although the SA-deficient plants show the same level of susceptibility to *R. solanacearum* as the wild type (Hirsch *et al.*, 2002; Hernández-Blanco *et al.*, 2007; Hanemian *et al.*, 2016), previous reports indicate that SA may participate in defense against this pathogen. For instance, RRS1-R-mediated defense in Arabidopsis ecotype Niederzenz-1 is orchestrated by SA (Deslandes *et al.*, 2002). Additionally, SA partly contributes to the enhanced tolerance to *R. solanacearum* observed in the Arabidopsis mutant *wat1* (*Walls are Thin1*) (Denancé *et al.*, 2013). Further, SA participates in defense of other plant species to *R. solanacearum*, such as in tomato-resistant varieties Hawaii 7996 and CRA 66 (Baichoo and Jaufeerally-Fakim, 2016), and in tobacco (Lowe-Power *et al.*, 2016; Liu *et al.*, 2017).

It has been previously reported that treatments with SA or its analog BTH [benzo(1,2,3)thiadiazole-7-carbothioic acid *S*-methyl ester] are potent activators of plant defenses against various pathogens both in leaves (Achuo *et al.*, 2002; Herman *et al.*, 2008; War *et al.*, 2011; Azami-Sardooei *et al.*, 2013; Bektas and Eulgem, 2015; Kouzai *et al.*, 2018) and in roots (Attard *et al.*, 2010; Chuberre *et al.*, 2018). We found that exogenous SA application results in a partial reversion of the *in vitro* root phenotypes caused by *R. solanacearum* infection (Fig. 5A), similar to that we observed after cytokinin treatment (Fig. 4A, B). Our results indicate that SA might also contribute to root defenses against *R. solanacearum* at early stages of infection.

Although evidence is still limited, our results point towards the existence of an SA–cytokinins crosstalk in Arabidopsis roots after infection, since *R. solanacearum*-triggered expression of the cytokinin marker *TCSn::GFP* is significantly reduced by SA depletion in the *eds16* mutant when compared with wild-type plants (Fig. 5B–D). These data demonstrate that in the context of root infection, SA levels affect cytokinin signaling and, in turn, cytokinin signaling could modulate SA levels, although evidence proving the effects of cytokinin on SA signaling is still limited in this system. This indicates that crosstalk between the cytokinin and SA pathways in response to pathogens could also take place in response to *R. solanacearum* in roots and might affect defense response outcomes. Whether cytokinins, SA, and their crosstalk have a more general role in immunity against root-invading pathogens will be interesting to explore in the future.

Together, our data demonstrate that cytokinins participate in defense against *R. solanacearum* and are involved in the early root phenotypes caused by the pathogen at early stages of infection. While it is known that cytokinin plays a very important role in defense against bacteria, fungi, or viruses (Clarke *et al.*, 1998a; Pogány *et al.*, 2004; Choi *et al.*, 2010; Großkinsky *et al.*, 2011; Argueso *et al.*, 2012), our findings highlight a novel role for cytokinin in root immunity. Defenses in the root remain vastly unexplored and our study adds evidence indicating that pathogen perception in the root activates cytokinin metabolism and signaling, which modulates plant immunity contributing to plant defense.

## Supplementary data

The following supplementary data are available at *JXB* online.

Fig. S1. Genome-wide association study experimental scheme.

Fig. S2. Zoom-in of SNP positions.

Fig. S3. Ecotype distribution based on root growth data after infection with *R. solanacearum.*

Fig. S4. Role of SA in *R. solanacearum* infection.

Fig. S5. Disease index scale of Arabidopsis plants after *R. solanacearum* infection.

Table S1. Ecotypes used in this study.

Table S2. Root growth data.

Table S3. Heritability rates.

Table S.4 Top SNPs.

Table S5. Gene Ontology (GO) analysis.

Table S6. Primers used for quantitative PCR.

eraa610_suppl_Supplementary_Figures_S1-S5

eraa610_suppl_Supplementary_Tables_S1-S6

## Data Availability

The data that support the findings of this study are available from the corresponding author, NSC, upon reasonable request.
