# Supplementary material for: A genome-wide association study reveals cytokinin as a major component in the root defense responses against Ralstonia solanacearum
Source: J Exp Bot. 2021 Jan 21;72(7):2727–40. doi: 10.1093/jxb/eraa610 (PMC8006551; doi:10.1093/jxb/eraa610)
Supplement: eraa610_suppl_Supplementary_Figures_S1-S5 [file eraa610_suppl_supplementary_figures_s1-s5.pdf]

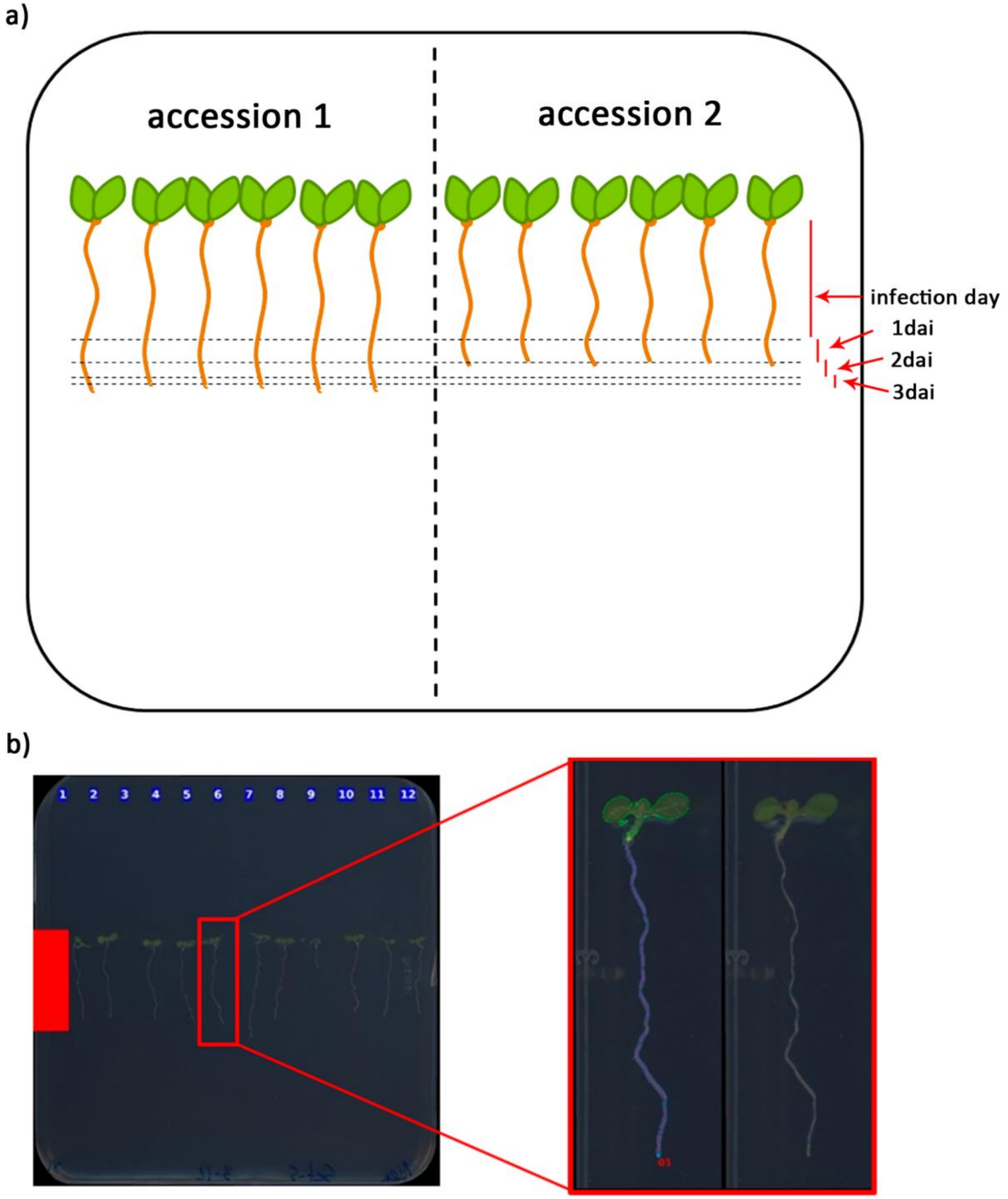

Figure S1: **GWAS planting model**. a) seven-day-old seedlings distribution from two different accessions after *R. solanacearum* infection. Twelve seeds per accession were sow, distributed between two MS medium (six seeds per MS- plate). **b)** BRAT software.

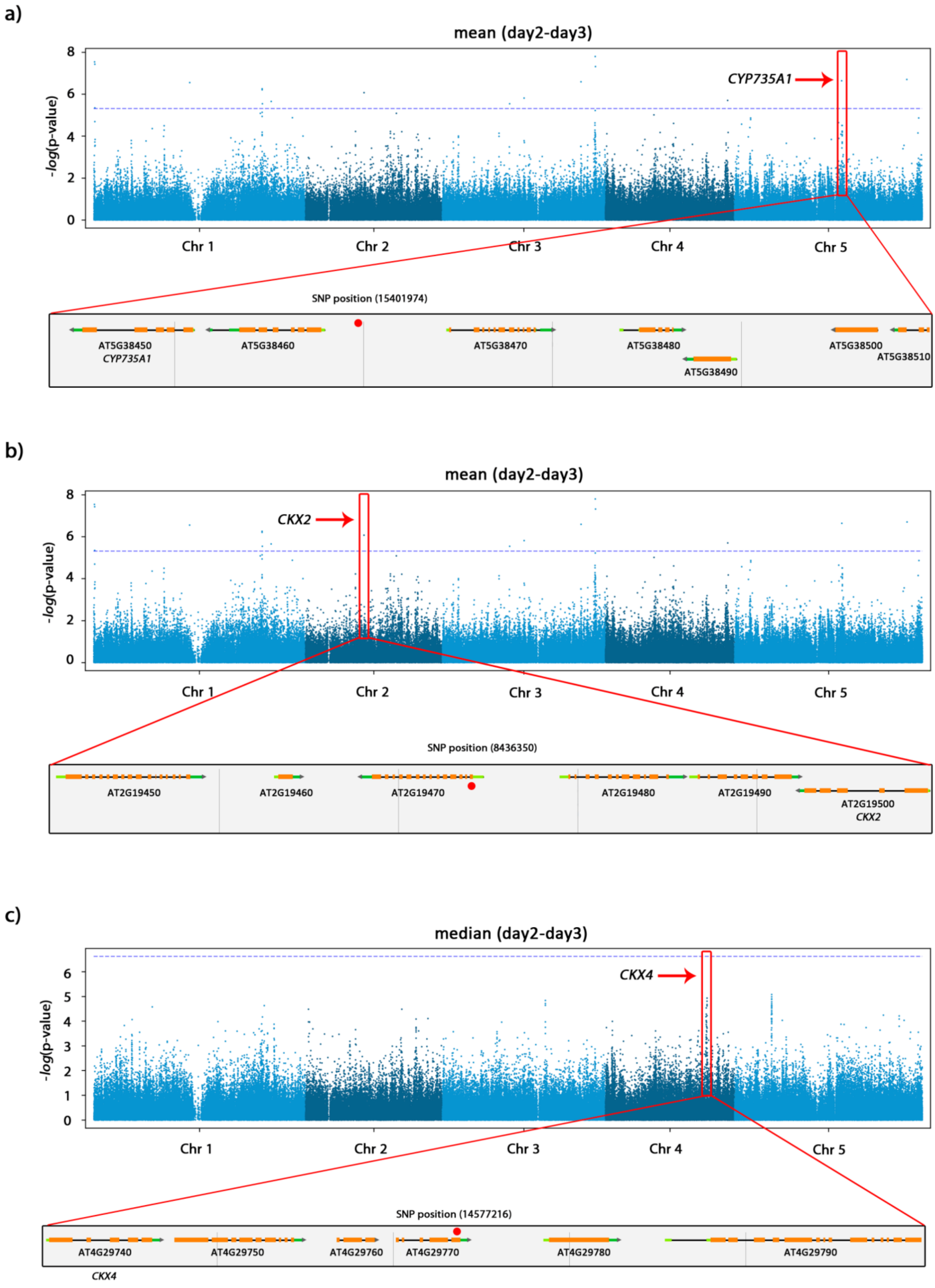

Figure S2: **Zoom in SNP position**. a) SNP 15401974 on mean relative root growth rate (day2-day3), and b) SNP 8436350 on mean relative root growth rate (day2-day3) and SNP 14577216 on median relative root growth rate (day2-day3).

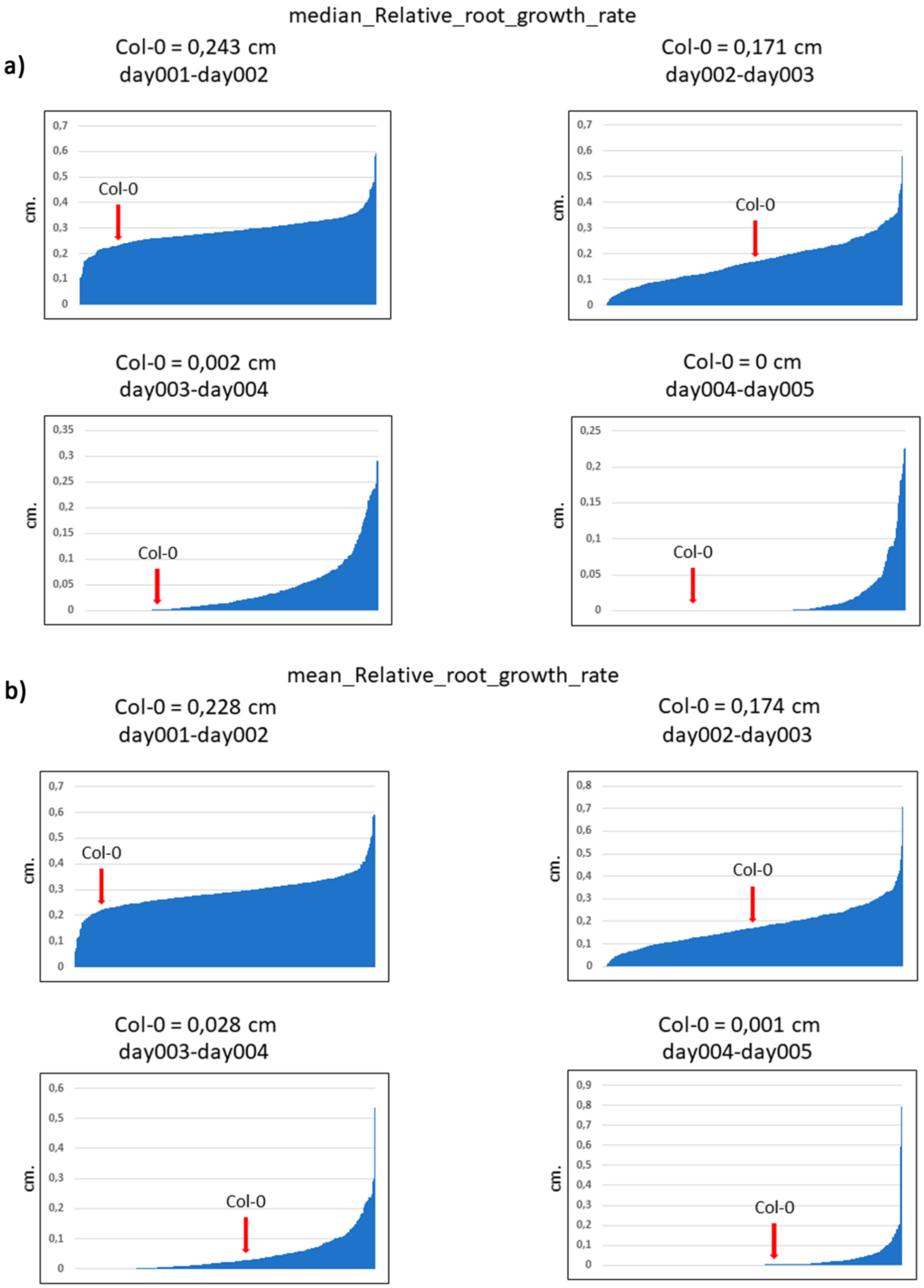

Figure S3: Col-0 ecotype distribution based on root growth after *Ralstonia solanacearum* infection. a) median relative root growth rate and b) mean relative root growth rate.

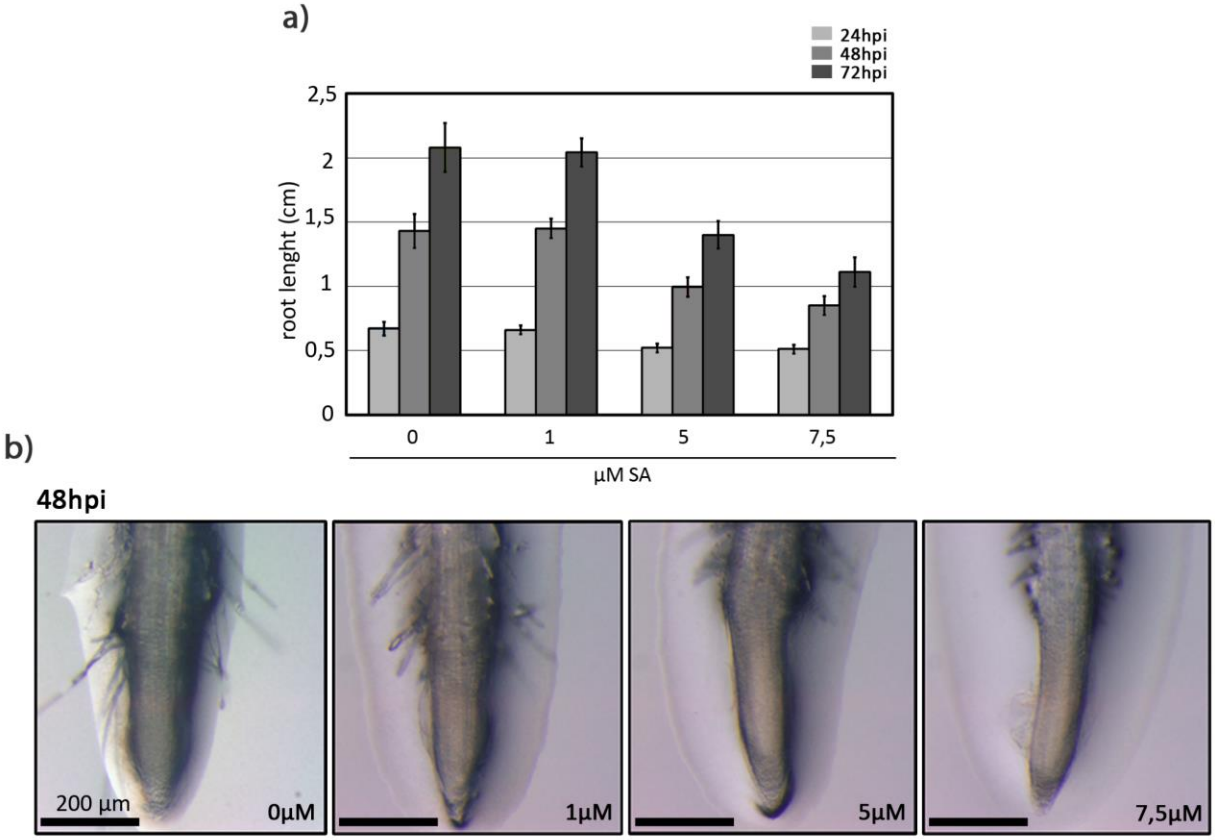

Figure S4: Role of SA in *R. solanacearum* infection. Seven-day-old Col-0. Seedlings were grown for 24 hours in MS medium supplemented with SA (0, 1, 5 and 7,5µM) and were inoculated with *R. solanacearum* or water; a) root lenght was measured in water control and b) root hair production under *R. solanacearum* infection at 48hpi.

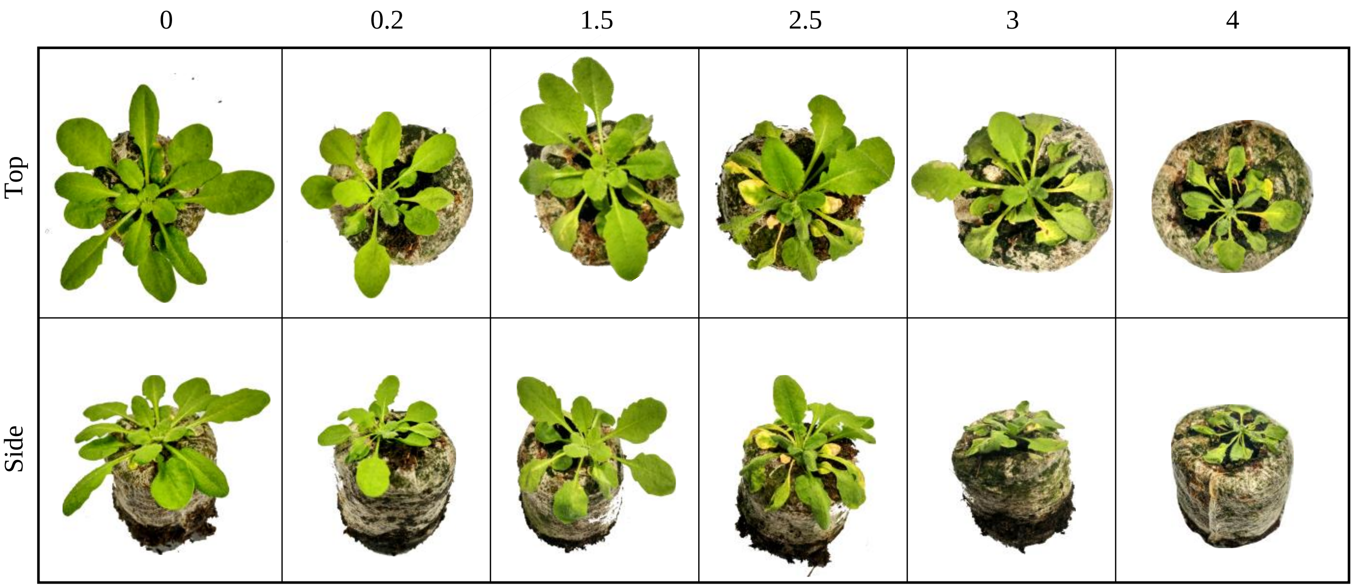

Figure S5: Disease index scale of Arabidopsis plants after *R. solanacearum* infection. A disease index scale was calculated based on symptoms ranging from 0 – 4, where 0 = no symptoms to 4 = completely wilted plant. Pictures were taken from the top (top) or side (bottom) of the jiffys at different d.p.i., when plants showed the specified symptoms showed on top.
